# The National DNA Data Bank of Canada: a Quebecer perspective

**DOI:** 10.3389/fgene.2013.00249

**Published:** 2013-11-20

**Authors:** Emmanuel Milot, Marie M. J. Lecomte, Hugo Germain, Frank Crispino

**Affiliations:** ^1^Groupe de Recherche PRIMUS, Faculté de Médecine, Université de SherbrookeSherbrooke, QC, Canada; ^2^Mt. Albert Science Centre, The Institute of Environmental Science and Research Ltd.Auckland, New Zealand; ^3^The Department of Forensic Science, School of Chemical Sciences, The University of AucklandAuckland, New Zealand; ^4^Département de Chimie, Biochimie et Physique, Université du Québec à Trois-RivièresQC, Canada

**Keywords:** DNA database, Canada, Québec, genetic engineering, forensic challenges

## Abstract

The Canadian National DNA Database was created in 1998 and first used in the mid-2000. Under management by the RCMP, the National DNA Data Bank of Canada offers each year satisfactory reported statistics for its use and efficiency. Built on two indexes (convicted offenders and crime scene indexes), the database not only provides increasing matches to offenders or linked traces to the various police forces of the nation, but offers a memory repository for cold cases. Despite these achievements, the data bank is now facing new challenges that will inevitably defy the way the database is currently used. These arise from the increasing power of detection of DNA traces, the diversity of demands from police investigators and the growth of the bank itself. Examples of new requirements from the database now include familial searches, low-copy-number analyses and the correct interpretation of mixed samples. This paper aims to develop on the original way set in Québec to address some of these challenges. Nevertheless, analytic and technological advances will inevitably lead to the introduction of new technologies in forensic laboratories, such as single cell sequencing, phenotyping, and proteomics. Furthermore, it will not only request a new holistic/global approach of the forensic molecular biology sciences (through academia and a more investigative role in the laboratory), but also new legal developments. Far from being exhaustive, this paper highlights some of the current use of the database, its potential for the future, and opportunity to expand as a result of recent technological developments in molecular biology, including, but not limited to DNA identification.

## The canadian national DNA data bank

At the time the UK launched its DNA database in 1995, the exonerations of two wrongly accused individuals (Morin case, 1985 and Milgaard case, 1969) and the implementation of the C-104 bill (to amend the Criminal Code and the Young Offenders Act) acknowledged the need for a similar requirement in Canada and initiated the creation of the Canadian National DNA database (NDDB) by the Identification Act (Law C-37 of Dec. 10th, 1998) (Curran, 1997).

Following a nation-wide consultation with various institutional bodies (such as the Privacy Commissioner, the Canadian Bar Association, and the Canadian Police association), to address ethical, legal, and social implications issues also tackled by the National Human Genome Research Institute's (NHGRI) during the human Genome Project, its operative use was launched immediately after the proclamation of the S-10 bill on June 2000. A number of amendments led the NDDB to store genetic traces collected at crime scenes in the Crime Scene Index (CSI) and, under court order, the DNA profiles of offenders serving any sentence of imprisonment, for various categories of offences designated in section 487.04 of the criminal code, in the Convicted Offenders Index (COI).

Under the supervision of the DNA Data Bank Advisory Committee, composed of seven authoritative personalities involved in forensic biology, human rights and laboratory management, the NDDB is operated by the Royal Canadian Mounted Police (RCMP) for the benefit of all law enforcement agencies in the country, be it federal (the RCMP), provincial [the Ontario Police force or Sûreté du Québec (SQ)] or urban (depending on the level of police a town has to deliver in regard to its population), as provided by the RCMP at provincial and urban levels if requested.

**Figure 1:**
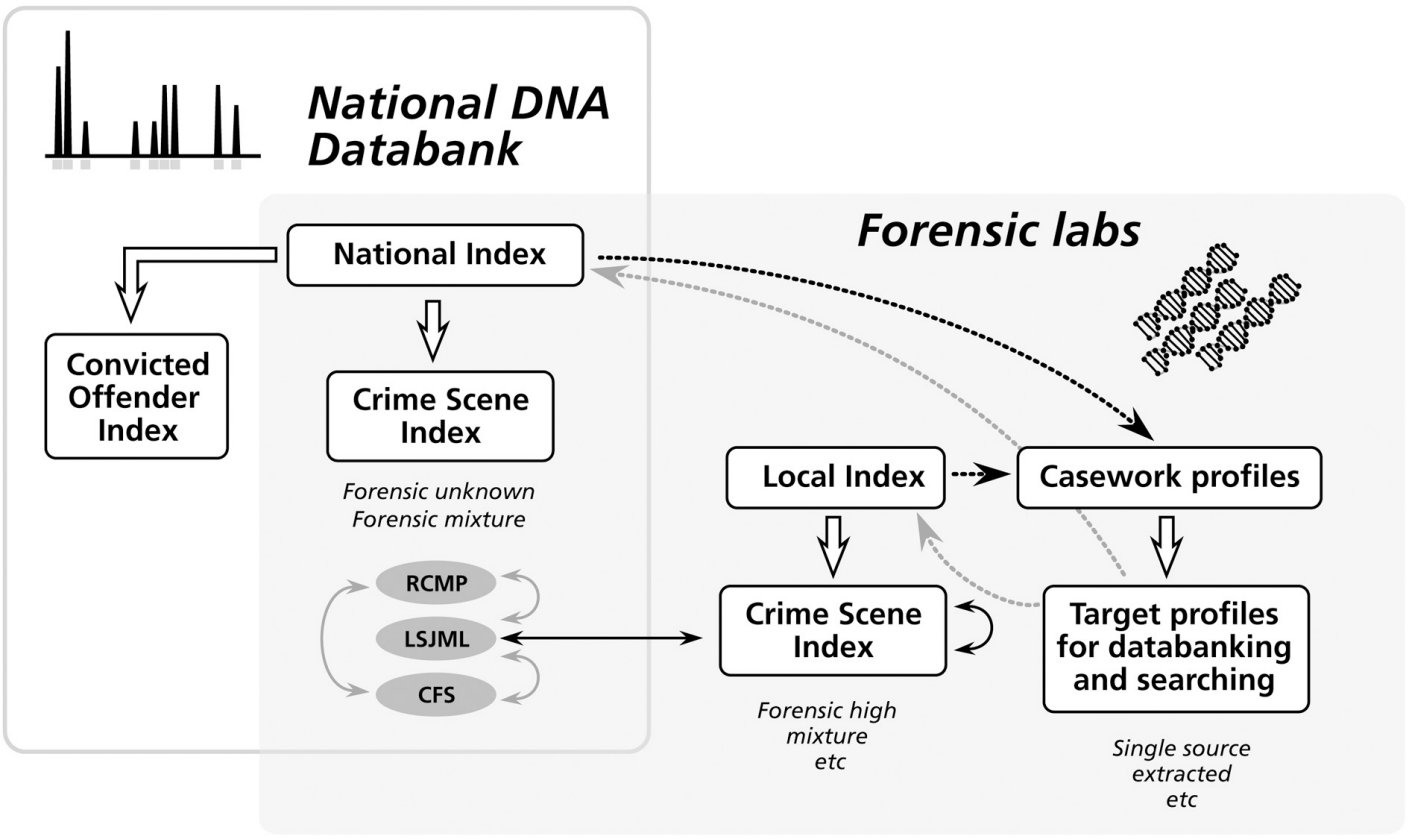
**The architecture of the *National DNA Data Bank in Canada* and its relationship to forensic laboratories**. The bank is a national repository composed of two indexes. The Convicted Offender Index is managed centrally at the national level, while the National Crime Scene Index (CSI-nat) is managed collectively by three forensic labs (RCMP, LSJML, and CFS), with each lab being responsible for the profiles generated under its jurisdiction. The CSI-nat allows for inter-jurisdictional comparisons of crime scene profiles (solid gray arrows). The Local Crime Scene Index (CSI-loc) corresponds to the databases maintained locally by forensic labs and containing DNA profiles that do not meet the criteria to be deposited in the NDDB (e.g., some complex mixtures). Local comparisons can be made both between profiles stored in the same CSI-loc and between profiles of the CSI-loc of a given lab and the portion of the CSI-nat managed by the same lab (solid black arrows; see Figure 2). Gray dotted arrows show the deposition of DNA profiles from caseworks into the national and local indexes, while black dotted arrows illustrate match information returned to the forensic labs.

The CSI is maintained by the RCMP labs, the Center of Forensic Sciences in Toronto (CFS^1^) and the Laboratoire de sciences judiciaires et de médecine légale du Québec in Montréal (LSJML^2^) (Figure 1). On July 15th, 2013, the COI contains more than 273,000 profiles, while the CSI is nearing 87,000^3^.

Based on 13 DNA markers, DNA profiles are managed and compared using the Combined DNA Index System (CODIS). On a yearly basis, a RCMP report on the management of the NDDB provides statistics, financial costs information, a user guide for the reader, as well as information on the changing legal frame of the database, under the auspices of the Advisory Committee (Police, 2012).

The Advisory Committee controls and actively searches and suggests legislative and regulatory changes. This transparency in the management of the NDDB is what leads to the efficiency of the Canadian system, qualified as having “an astonishing degree of consistency in sampling regimes throughout the history of the Canadian DNA database.” (Walsh, 2009). Using the ratio of hits over the product of NC, (N being the numbers of profiles in the COI and C the numbers of profiles in the CSI), to assess the efficiency of DNA databases between four western countries (USA, the United Kingdom, the Netherlands, and New Zealand), the performance of the NDDB ranks just below New Zealand and is quite good, accounting for the lower proportion of the population being present in the database (0.5% for Canada instead of 2.1% for New Zealand). In regards to the public perception of civil rights, it could easily be deemed highly efficient. At least “Canada had a well-understood and effectively resourced concept of operation in place prior to the initiation of databasing” (Walsh, 2009). On such ground, Canada seems better prepared than many other countries to tackle new challenges facing forensic DNA identification.

## The LSJML databanking strategy

As with other DNA databanks, the NDDB holds key figures to address interpretation issues (Foreman et al., 2003; Dror and Hampikian, 2011) such as low copy numbers (LCN) (Lowe et al., 2002; Phipps and Petricevic, 2007), mixed samples (Bill et al., 2005; Curran, 2008), and familial searches (Bieber et al., 2006; Reid et al., 2008; Miller, 2010; Murphy, 2010; Gershaw et al., 2011; Meyers et al., 2011; Pham-Hoi et al., 2013). While addressing the issue of familial searches is not yet on the agenda, as it would require changes to the Canadian legislation, LCN has become a routine challenge faced by forensic labs nationwide. Indeed, due to technological improvements, the detection of ever-smaller traces of DNA is now possible (Kayser and de Knijff, 2011). However, because of stochastic effects (drop-outs, drop-ins), this comes at the cost of lower repeatability and overall completeness of genetic profiles recovered from small quantities of DNA. This problem is made worse with mixtures owing to competitive amplification. Deconvoluting the information and sorting out the alleles of each contributor in a mixture can become hard to achieve even in simpler cases such as a mixed profile from two contributors. As a consequence of these new challenges, forensic laboratories, and the database managers may use various criteria to limit the deposition of mixed or partial profiles into the NDDB. For instance, the NDDB will only accept mixtures with data for *L* STR loci, where 9 ≤ L ≤ 13 with a maximum number of loci exhibiting more than two alleles equal to *L*−7, and with no more than five alleles per locus. Although STRs exhibit very high level of polymorphism enabling high discriminatory power, they are subject, like any amplification-based markers, to the presence of polymorphisms within the primer binding site which results in lack of amplification or so-called drop-out alleles (or null alleles). The impact of such result has been well documented (Haned et al., 2011) and probabilistic methods can be used to account for drop-in and drop-out alleles (Gill et al., 2012).

With these managerial constraints, the development of statistical methodologies allowing more formal quantitative comparisons of casework profiles to DNA databanks is required. In the meantime, the LSJML has developed an innovative investigative strategy to increase the use of partial profiles from LCN or complex mixtures in their search for matches in the databanks, relying on two complementary practices.

Elaborated interpretation and databanking guidelines at LSJML allow the specific extraction of the relevant genetic information contained in single-source or mixed profiles for databank searches for intelligence purposes (Noël et al., 2009). For instance, the flagging of alleles as “obligate” or “non-obligate” in queries sent to the NDDB allows filtering out considerably the potential matches, limiting them to a subset of possible matches that is consistent (see section Challenges of the LSJML model and research prospects) with all the information available for the casework. For example, this procedure is used to separate alleles that are likely to come from the putative aggressor in intimate swabs from the victim of a sexual assault—i.e., alleles that must be included in any candidate match returned by the NDDB—from alleles of less certain origin (e.g., alleles of the victim potentially shared with the aggressor) that need not be present in the candidate profile. More generally, this approach is valid for any mixture related to any type of infraction where some of the alleles are more likely than others to come from the offender(s). Another option is to eliminate alleles from a person whose DNA profile is known from other traces obtained for the same casework (e.g., victim, witness or single-source unknown), or those that would imply either highly unbalanced peak heights of a contributor to a mixture or dropouts when it is not a reasonable possibility based on statistical data. It is up to the reporting scientist to check the relevance of the hypothesis with his/her scientific investigation of the case.

**Figure 2:**
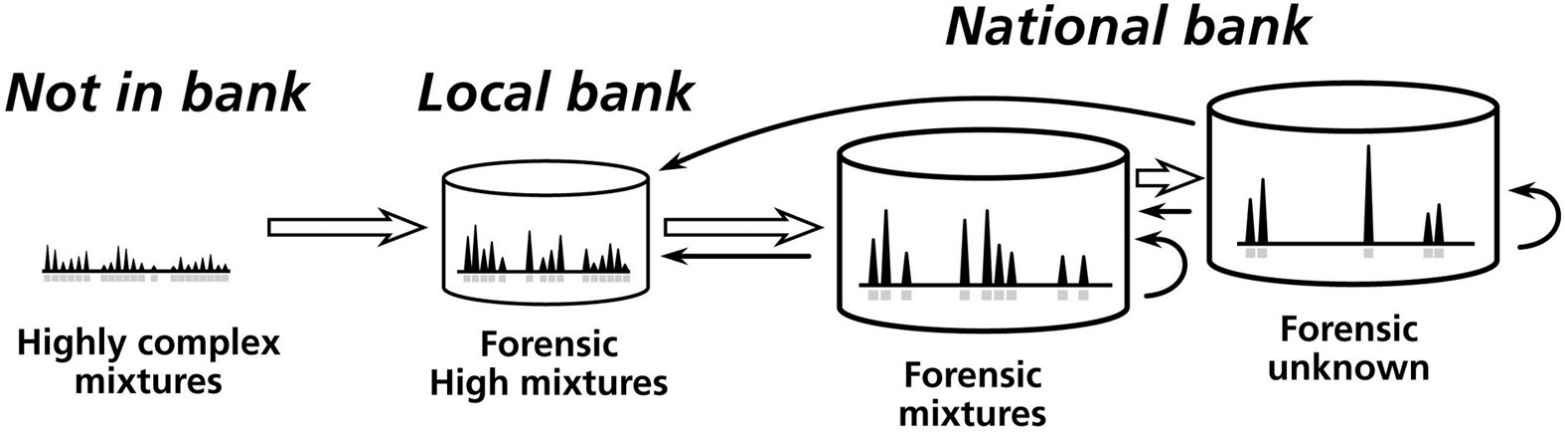
**The processes of mixture databanking and comparison at LSJML**. The complexity of profiles decreases from left to right, i.e., from highly complex mixtures that cannot be deposited as is in databank to single-source profiles stored under the “forensic unknown” index. “Forensic high mixture” and “Forensic mixture” are two intermediate indexes, respectively stored at the local (LSJML) and national (NDDB) levels. These three indexes composed the Crime Scene Index (CSI; see Figure 1). Open arrows show how a mixture can switch category when alleles are removed from it (e.g., alleles of low intensity or from a known contributor; see section The LSJML databanking strategy). Solid arrows indicate how profiles from the different indexes are compared in search for matches.

The second aspect of the LSJML strategy is the maintenance of its own local database (also hosted in the CODIS system) where complex mixtures that do not meet the NDDB criteria can be deposited, namely in the “Forensic High Mixture” index, for comparison with other local casework profiles (Figures 1, 2). In addition, the local database allows searching for matches using more loci, i.e., up to 15 at the LSJML operational setup instead of the 13 CODIS loci in the NDDB. Finally, mixed strategies are authorized whereby a full mixture can be deposited into the local database while a subset of its alleles (a “submixture”) is sent to the NDDB. Thus, matches can potentially occur at the local level between the whole mixture kept as a “backup” and pure or mixed profiles from other caseworks. This can be especially useful when deconvolution is difficult so that there is much uncertainty around which alleles should be sent at the NDDB.

## Challenges of the LSJML model and research prospects

While the LSJML model provides great flexibility in maximizing the number of matches, it also raises legitimate questions about potential biases that may arise from its databanking strategy (Lynch, 2003; Dror et al., 2006; Dror and Hampikian, 2011).

Aware of it, the LSJML has adopted different strategies to assess their importance and limit them. These range from operational rules to current and prospective research projects. First, the LSJML does not declare a match as valid as soon it occurs (except when both the target and the candidate are single-sourced and complete). Thus, once a match between a target profile and a candidate profile in the NDDB has occurred, the LSJML scientist must assess its validity. The procedure involves an evaluation of the candidate profile using the original electropherogram from which the target profile was extracted, statistical data on peak height balance and drop-outs, as well as other profiles from the casework. This is the step where consistence with all the information available for the casework is evaluated. In addition, the validity of the match must also be confirmed by one of the two local scientists managing the databank.

Second, because the above procedure may limit but not completely eliminate fortuitous (wrong) matches, the opinion on evidential weight (Providers, 2009) is based on standard statistical approaches such as the probability of exclusion or likelihood ratios performed on the whole mixture, and not on extracted elements, except when a major profile can clearly be extracted using strict deconvolution rules.

Third, the LSJML has been proactive in challenging the validity of its own strategy with respect to biases or invalid match generation by undertaking a number of quantitative statistical evaluations. It is worthy to note that the databanking and match review strategies for targets arising from mixtures of various levels of complexity generate valid candidate matches in comparable proportions to single-source targets (for which match validity is automatic), with similar levels of effort (i.e., working time required to evaluate the matches) see (Noël et al., 2009) and (Lavergne et al., 2008) for details. For instance, less than 12% of candidate matches produced with the Forensic Unknown and Forensic Mixture indexes (Figure 2) are rejected with a “no match” disposition after review. One critical aspect is that mixtures must be of good quality, namely show good peak intensities. Moreover, the LSJML has begun to perform experimental tests by searching two-person mixtures with up to 13 mixed loci against the Florida data bank constituted of nearly 500,000 convicted offender profiles. Because of the geographical (~2000 km) and country barriers between Québec and Florida, it is expected that almost any eventual match would be fortuitous. Corroborating above conclusions, these mixtures did not return more candidate matches than less complex ones. Moreover, all candidate matches were rejected independently (i.e., not in concert) by four reporting scientists. Finally, the lab, in collaboration with others, is presently evaluating an alternative to the current selection procedure for uploading mixtures to the NDDB. The new approach would be based on the number of expected matches accounting for the COI size and is implemented in the CODIS Match Estimator® module.

At this time, open discussion between LSJML and academic partners to better assess the potential hazards of inducing these databanking policies with respect to confirmations bias are currently underway. Nevertheless, an understanding of this strategy with respect to a possible future goal toward forensic intelligence should be kept in mind (Ribaux et al., 2006; Pham-Hoi et al., 2013). On the other hand, limiting decisions into whether identification was correct or not by only using pure profiles may provide a sense of security. However, this also leads to the restricted use of the information available, with potentially pertinent information discarded when solving everyday crimes. It is currently unclear what the consequences of refusing to tackle these issues will have on victims, and consequently on justice, who also has a validating role to play in this area.

Nevertheless, a fine-tuned approach, specific to the various types of casework (sexual assault, homicide, burglary, high-volume crimes, etc.) definitely needs to be addressed to better, and more rigorously, assess the consequences these changes will have on the whole process of identification.

## Beyond the present DNA practice

Notwithstanding these innovative practices and the relevant interpretation process to be developed being a sign of academic-practitioner joint effort, the development of STR mixture analysis and databank searching will eventually reach its limit impeding further improvements owing to the inherent limitations of using small sets of markers (typically < 20 for STR) typed by technologies that do not permit to separate DNA from different cells found in the same trace (with the exception of differential extraction of semen DNA). Ultimately, substantial increase in the power of mixture analysis will come from newer technologies such as single nucleotide polymorphisms (SNPs) (Daniel and Walsh, 2006; Kidd et al., 2006; Sanchez et al., 2006; Fang et al., 2009; Pakstis et al., 2010; Voskoboinik and Darvasi, 2011) or single-cell sequencing (Hanson and Ballantyne, 2005). Repositories like the NDDB will need to adapt to these forthcoming innovations in a way that permit forensic labs to benefit from the full power of these new tools for match searching, but without compromising on the usefulness of the STR information accumulated since their creation.

Other advances in the biological sciences, not strictly depending on the NDDB itself, could benefit from the advice/input/review of the Advisory committee to pave the way for a new forensic dimension (Daniel and Walsh, 2006; Kidd et al., 2006). Research into fields such as ancestry informative markers (AIMs) (Lao et al., 2008; Kersbergen et al., 2009; Kosoy et al., 2009; Liu et al., 2009), proteomics (Kool et al., 2007; Lecomte et al., 2013), genome/marker based phenotyping (Sulem et al., 2008; Liu et al., 2009; Zubakov et al., 2010; Walsh et al., 2011), framing the input of DNA to forensic intelligence (Jobling and Gill, 2004; Ribaux et al., 2006; Bond, 2007; Roman et al., 2009; Wilson et al., 2011), and the incoming lab-on-a-chip involvement of crime scene (Batt et al., 2009; Bell, 2011). All these fields belong to a still-debated investigative process (Kaye, 2007) opposed to the claim for a strict separation of laboratories from the law enforcement system (Nrc, 2009). Box 1 presents two examples of techniques that could eventually be used in forensic sciences on a case-by-case basis. One of them, forensic proteomics, does not directly assist to the evolution of the NDDB. However, the power of these new tools to address personal characteristics of human beings, could lead to an ethical position being taken by the Advisory Committee, which could impact the future developments of the data bank.

Box 1 **Beyond present DNA practices**

1) Going further with genomics

While it was initially believed PCR would be capable of solving the challenges encountered from analysing LCN samples, difficulties associated with interpretation lessens its pragmatic use in forensic casework (Gill et al., 2000; Kloosterman and Kersbergen, 2006; McCartney, 2008; Budowle et al., 2009) (see also LCN DNA Review at http://www.mccannfiles.com/id190.html). In addition, the use of a limited set of markers, as is the case today, restricts the potential discriminative power that could be accessed if using full genome sequences.

Single-cell genome sequencing is a rapidly improving technology with one of many applications including the detection of somatic intra-individual variation in cancer patients (Navin et al., 2011). Initially applied to small prokaryotic genomes (Stepanauskas and Sieracki, 2007), recent advances in next-generation sequencing have enabled the coverage of 93% of the much larger human genome from a single human cell (Zong et al., 2012). This not only allows for the identification of SNPs and LCN variation (Zong et al., 2012) but also genomic structural variation and somatic mutations that give rise to intra-individual genetic variation (O'Huallachain et al., 2012). With as many as 2500 genomic structural variations and three million SNPs (Abecasis et al., 2010) occurring between two unrelated individuals, the discriminative power of this technique could even allow for identical twins to be differentiated.

Full genome sequencing would permit the use of a much wider range of genomic polymorphism to convict or exonerate persons of interest. Although currently cost prohibitive, and against the current ideology that the use of anonymous loci is preferable, the dwindling cost associated with genome sequencing may enable their use in a foreseeable future.

2) Adding transcriptomics and proteomics to the forensic toolbox ?

With research carried out in the fields of transcriptomics and proteomics, opportunities are emerging to develop and add to the already existing genomic platforms.

Evidence of physical abuse is often left of the skin of victims, with bruising found to be the most common form of injury (Dye et al., 2008; Pierce et al., 2010; Jackson et al., 2012). The ability to accurately and reliably determine the age of a bruise, in living individuals, is currently lacking. If possible, this could provide vital evidence to legal cases of suspected physical abuse. In cases where multiple bruises are present on the body of a victim, providing evidence that the injuries were inflicted on separate occasions could have important medico-legal significance.

Building a human proteome map of protein markers present in unbruised skin, as well as bruised skin, and analysing changes in protein expression levels as a bruise evolves, could help to achieve these goals (Lecomte et al., 2013).

## Conclusion

As exciting projects make their way in the field of molecular biology, real challenges also lie in the realm of forensic science, giving new impedimenta to forensic DNA and, raising obvious ethical, social, and economic questions. Nevertheless, the inescapable drive toward DNA intelligence and laboratory miniaturization, and the projection on the crime scene, could underline the need for a better scientific support of the crime scene officers present at the start of the forensic process.

As commissioner Paulson of the NDDB wrote in the last annual report, “the NDDB operates within a diverse environment that must consider scientific advancements, privacy rights, and changing legislation.” In regards to the building up of the NDDB and the wisdom of its Advisory committee, an optimistic future for the scientific support of the Canadian law and justice systems is anticipated.

## Author contribution

Emmanuel Milot is post-PhD researcher in biology and consultant for the LSJML and the RCMP laboratories. His research interest addresses two domains: the use of genetics to study human and animal population dynamics and the causes of phenotypic variations between individuals.

Marie M. J. Lecomte has recently submitted her PhD employing proteomic techniques to determine the age of bruises in living individuals.

Hugo Germain is professor of biochemistry, head of chair on vegetal immunity, in charge of human DNA identification course at the forensic curriculum at the UQTR.

Frank Crispino is criminalist with a PhD degree from the University of Lausanne (Switzerland) and a former criminal and counter-terrorist investigation commander in the French Gendarmerie. He is now serving as professor in forensic science at the UQTR.

Although this article is a common elaborated paper, the order of appearance reflects their relative inputs in the paper, the corresponding author being, moreover, in charge of coordination.

### Conflict of interest statement

The authors declare that the research was conducted in the absence of any commercial or financial relationships that could be construed as a potential conflict of interest.

## Footnotes

^1^http://www.mcscs.jus.gov.on.ca/english/centre_forensic/CFS_intro.html.

^2^http://www.securitepublique.gouv.qc.ca/lsjml.html.

^3^http://www.rcmp-grc.gc.ca/nddb-bndg/stats-eng.htm.
